# Contrast sensitivity in glaucoma patients with visual field defects at different locations

**DOI:** 10.1038/s41598-022-27262-z

**Published:** 2023-01-02

**Authors:** Ji Yong Jang, Eun Ji Lee

**Affiliations:** Department of Ophthalmology, Seoul National University College of Medicine, Seoul National University Bundang Hospital, 82, Gumi-ro, 173 Beon-gil, Bundang-gu, Seongnam, Gyeonggi-do 463-707 Korea

**Keywords:** Optic nerve diseases, Glaucoma

## Abstract

Contrast sensitivity (CS) is closely associated with vision-related quality of life in glaucoma patients. This cross-sectional study was performed to determine the relationship between CS and visual field (VF) sensitivity (VFS) according to the hemifield location of VF defects in 238 patients with primary open-angle glaucoma (POAG) who underwent 24-2 standard automated perimetry and CS measurement on the same day. Of the 238 eyes, 83, 58, and 47 eyes had VF defects in the superior, inferior and both hemifields, respectively, and 50 had no VF defects in either hemifield. Significant correlations between CS and VFS in all sectors were observed in the entire cohort (*R*^*2*^ = 0.103–0.315, all *P* < 0.001). However, CS poorly represented VF defects in eyes with single superior (*R*^*2*^ = 0.037–0.086) or inferior (*R*^*2*^ = 0.107–0.222) hemifield defects. Eyes with bi-hemifield VF defects showed significant correlations between VFS and CS at 0.3 m in all sectors (*R*^*2*^ = 0.200–0.406), but the correlation between VFS and CS at 5 m was significant only in the inferior sector (*R*^*2*^ = 0.224–0.348 vs. 0.081–0.126 in the inferior and superior sectors, respectively). Correlations between CS and VFS were dependent on CS distances and the presence of bi-hemifield VF defects. Although CS may not correlate with VFS in eyes with single-hemifield VF defects, it may reflect visual disability in eyes with bi-hemifield defects.

## Introduction

Assessment of visual function is an important process in glaucoma evaluation because functional deficits associated with glaucomatous damage are closely linked to patients’ quality of life (QOL) and activities of daily living^1–5^. Visual field (VF) tests, such as standard automated perimetry, are considered the standard method for evaluating visual function in patients with glaucoma. Glaucoma patients, however, describe their decreased vision as poor image quality or a need for more light, rather than a limit in VF scope^6^, suggesting that VF tests have a limited ability to determine the level of disability experienced by glaucoma patients in their daily lives. In addition, standard VF tests have drawbacks, including the attentiveness required of patients due to the long testing time, which frequently results in poor test reliability.

Contrast sensitivity (CS) is the ability to distinguish between increments of light and dark^7^. Patients with a low CS may have difficulty distinguishing the boundaries of objects, especially in the dark. CS has been found to correlate with VF sensitivity (VFS)^1,2,8^ in glaucoma patients, as well as being significantly associated with vision-related QOL^3,9^. The shorter testing time avoids the fatigue effect that could confound results.

It is suspected that visual disability is affected more by the location of the VF defect^10–12^ rather than by the severity of the VF damage itself^12^. Given the relationship between CS and VFS, CS may represent different fields of glaucomatous damage, with the usefulness of CS differing according to the location of VF damage. The present study therefore analyzed the relationship of CS with the location and pattern of VF damage in glaucomatous eyes.

## Results

This study included 238 eyes of 238 subjects; their demographic and clinical characteristics are shown in Table 1. All CS and VFS parameters well correlated with best-corrected visual acuity (BCVA, Supplemental Table 1).

**Table 1:** Clinical characteristics of participants.

Variables	
Age, years	57.2 ± 12.6
Sex (female/male), n	102/136
BCVA, LogMAR	0.06 ± 0.18
Axial length, mm	25.07 ± 1.75
Central corneal thickness, μm	541.9 ± 38.3
Intraocular pressure, mmHg	12.5 ± 2.7
Mean CS at 0.3 m, (log unit)	1.00 ± 0.41
Mean CS at 5 m, (log unit)	1.16 ± 0.36
VF MD, dB	− 5.54 ± 5.62
VF PSD, dB	5.90 ± 4.11

BCVA = best corrected visual acuity; LogMAR = Logarithm of the Minimum Angle of Resolution; CS = contrast sensitivity; VF = visual field; MD = mean deviation; PSD = pattern standard deviation.

Of the 238 eyes, 83, 58, and 47 had VF defects in the superior, inferior and both hemifields, respectively, whereas the remaining 50 eyes had no VF abnormalities (preperimetric glaucoma). A comparison of the clinical characteristics of these four groups showed that age differed significantly, with subjects having eyes with bi-hemifield VF defects being the oldest, followed by subjects with eyes having inferior, superior and no hemifield defects (*P* = 0.004) (Table 2). As expected, BCVA, VF MD and VF PSD differed among the four groups (all *P* ≤ 0.001), whereas there were no significant differences in sex distribution, AXL, CCT and intraocular pressure (IOP).

**Table 2:** Comparison of the clinical characteristics in the POAG eyes with visual field defects in various hemifields.

	Superior hemifield defect, A (n = 83)	Inferior hemifield defect, B (n = 58)	Bi-hemifield defect, C (n = 47)	Preperimetric (no VF defect), D (n = 50)	*P*	*Post-hoc*^†^
Age, years	53.5 ± 12.4	58.7 ± 12.0	60.9 ± 12.8	54.4 ± 12.1	**0.004**	A = D ≤ B ≤ C
Sex (female/male), n*	42/41	20/38	20/27	20/30	0.277	
BCVA, LogMAR	0.04 ± 0.17	0.07 ± 0.18	0.14 ± 0.22	− 0.01 ± 0.12	**0.001**	D ≤ A ≤ B ≤ C
Axial length, mm	25.24 ± 1.82	24.97 ± 1.67	24.70 ± 1.66	25.25 ± 1.78	0.318	
Central corneal thickness, μm	541.8 ± 40.2	544.4 ± 39.71	542.5 ± 37.3	538.7 ± 34.9	0.905	
Intraocular pressure, mmHg	12.4 ± 2.4	12.5 ± 2.7	12.1 ± 3.2	12.9 ± 2.7	0.572	
VF MD, dB	− 5.31 ± 3.56	-4.74 ± 3.86	− 12.15 ± 6.97	− 0.62 ± 1.10	** < 0.001**	C < A = B < D
VF PSD, dB	6.81 ± 4.10	5.90 ± 3.67	8.55 ± 3.33	1.87 ± 1.66	** < 0.001**	D < B = A < C

*Comparison was performed using Chi-square test. Other comparisons were performed using one-way analysis of variance.

^†^Tukey-b’s post-hoc test.

Significant values are shown in bold.

POAG = primary open-angle glaucoma; BCVA = best-corrected visual acuity; LogMAR = Logarithm of the Minimum Angle of Resolution; VF = visual field; MD = mean deviation; PSD = pattern standard deviation.

CS was most severely reduced in the eyes with bi-hemifield VF defects, followed in order by the eyes with inferior and superior hemifield VF defects (Table 3). VFS was lowest in the eyes with bi-hemifield defects, followed in order by eyes with superior or inferior hemifield defects (Table 3).

**Table 3:** Comparison of the contrast sensitivity and visual field sensitivity in the POAG eyes with visual field defects in various hemifields.

	Superior hemifield defect, A (n = 83)	Inferior hemifield defect, B (n = 58)	Bi-hemifield defect, C (n = 47)	Preperimetric (no VF defect), D (n = 50)	*P*	Post-hoc*
**CS**						
CS 0.3 m	1.05 ± 0.39	0.92 ± 0.37	0.77 ± 0.38	1.21 ± 0.38	** < 0.001**	C < B = A ≤ D
CS 5 m	1.17 ± 0.36	1.14 ± 0.35	1.02 ± 0.41	1.31 ± 0.28	**0.001**	C ≤ B ≤ A < D
**VFS (dB)**						
Total 24-2 VF	28.10 ± 1.95	27.76 ± 2.43	23.69 ± 4.00	30.22 ± 1.30	** < 0.001**	C < B = A < D
Sup 24-2 hemifield	25.22 ± 3.19	28.30 ± 2.29	22.21 ± 4.92	29.70 ± 1.44	** < 0.001**	C < A < B < D
Inf 24-2 hemifield	29.61 ± 1.92	26.97 ± 2.97	24.41 ± 3.94	30.65 ± 1.29	** < 0.001**	C < B < A < D
Central 10° VF	29.66 ± 2.00	29.79 ± 2.19	25.86 ± 3.94	32.03 ± 1.20	** < 0.001**	C < B = A < D
Sup central 10° hemifield	25.68 ± 6.64	30.18 ± 2.18	23.07 ± 6.88	31.62 ± 1.34	** < 0.001**	C < A < B = D
Inf central 10° hemifield	31.19 ± 1.89	29.14 ± 2.78	26.77 ± 4.10	32.37 ± 1.19	** < 0.001**	C < B < A < D
Central 5° VF	30.16 ± 2.25	30.59 ± 2.03	26.64 ± 4.34	32.65 ± 1.11	** < 0.001**	C < B = A < D
Sup central 5° hemifield	25.78 ± 6.63	30.18 ± 2.22	22.84 ± 7.39	31.66 ± 1.34	** < 0.001**	C < A < B = D
Inf central 5° hemifield	31.38 ± 1.92	29.08 ± 2.85	26.82 ± 4.57	32.49 ± 1.19	** < 0.001**	C < B < A < D

*Tukey-b’s post-hoc test.

Significant values are shown in bold.

POAG = primary open-angle glaucoma; CS = contrast sensitivity; VFS = visual field sensitivity; VF = visual field; Sup = superior; Inf = inferior.

**Figure 1:**
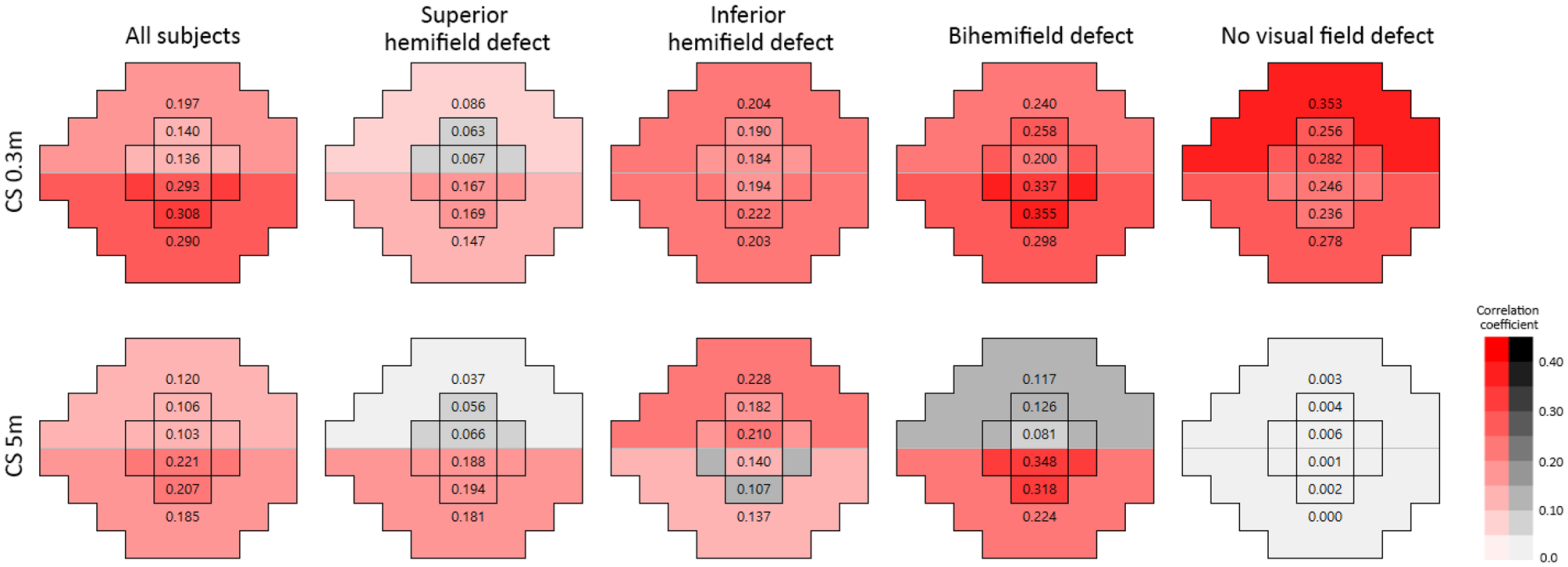
Correlations between contrast sensitivity and visual field sensitivities in various sectors according to the location of visual field defect. *Red* and *gray scales* were determined based on the correlation coefficient (the number in each sector), with *red scale* indicating statistically significant correlations. CS = contrast sensitivity.

After adjusting for subject age, significant correlations were observed between all CS and VFS parameters, including in the entire cohort (all *P* < 0.001, Table 4). Evaluation of the correlations between CS and VFS in various sectors of eyes in the four groups subdivided by location of VF defects showed overall good correlations in most sectors (Table 5). Exceptions, however, were observed, depending on the location of VF defects. For example, in eyes with superior hemifield defect, CS at 0.3 m did not correlate significantly with superior central 5° and 10° hemifield sensitivities, and CS at 5 m did not correlate significantly with superior central 5° and 10° hemifield sensitivities or with overall superior hemifield sensitivity. In eyes with inferior hemifield defect, CS at 5 m did not correlate significantly with inferior central 10° hemifield sensitivity. In the eyes with bi-hemifield defects, CS at 5 m did not correlate significantly with the superior central 5° and 10° and superior hemifield sensitivities. In eyes without VF defects, only CS at 0.3 m correlated significantly with VFS in all sectors, whereas CS at 5 m did not significantly correlate with VFS in any sector. The results shown in Tables 4 and 5 are illustrated in Fig. 1.

**Table 4:** Correlations between the visual field sensitivities in various sectors and the contrast sensitivity in POAG eyes after adjusting age.

VFS (dB)	CS 0.3 m		CS 5 m	
	*R*^*2*^	*P*	*R*^*2*^	*P*
**Total 24-2 VF**	0.296	** < 0.001**	0.196	** < 0.001**
Sup 24-2 hemifield	0.197	** < 0.001**	0.120	** < 0.001**
Inf 24-2 hemifield	0.290	** < 0.001**	0.185	** < 0.001**
**Central 10° VF**	0.315	** < 0.001**	0.229	** < 0.001**
Sup central 10° hemifield	0.140	** < 0.001**	0.106	** < 0.001**
Inf central 10° hemifield	0.308	** < 0.001**	0.207	** < 0.001**
**Central 5° VF**	0.290	** < 0.001**	0.221	** < 0.001**
Sup central 5° hemifield	0.136	** < 0.001**	0.103	** < 0.001**
Inf central 5° hemifield	0.293	** < 0.001**	0.221	** < 0.001**

Bonferroni correction was used for multiple comparisons. *P* value < 0.0083 (0.05/6) was considered significant.

Statistically significant values are shown in bold.

POAG = primary open-angle glaucoma; CS = contrast sensitivity; VFS = visual field sensitivity; VF = visual field; Sup = superior; Inf = inferior.

**Table 5:** Correlations between the contrast sensitivity and visual field sensitivities in various sectors according to the location of visual field defect in POAG eyes.

VFS (dB)	Superior hemifield defect (n = 83)				Inferior hemifield defect (n = 58)				Bi-hemifield defect (n = 47)				Preperimetric (no VF defect) (n = 50)			
	CS 0.3 m		CS 5 m		CS 0.3 m		CS 5 m		CS 0.3 m		CS 5 m		CS 0.3 m		CS 5 m	
	*R*^*2*^	*P*	*R*^*2*^	*P*	*R*^*2*^	*P*	*R*^*2*^	*P*	*R*^*2*^	*P*	*R*^*2*^	*P*	*R*^*2*^	*P*	*R*^*2*^	*P*
**Total 24-2 VF**	0.157	** < 0.001**	0.158	** < 0.001**	0.232	** < 0.001**	0.202	** < 0.001**	0.301	** < 0.001**	0.196	**0.002**	0.339	** < 0.001**	0.001	0.869
Sup 24-2 hemifield	0.086	**0.007**	0.037	0.083	0.240	** < 0.001**	0.228	** < 0.001**	0.240	**0.001**	0.117	0.019	0.353	** < 0.001**	0.003	0.699
Inf 24-2 hemifield	0.147	** < 0.001**	0.181	** < 0.001**	0.203	** < 0.001**	0.137	**0.004**	0.298	** < 0.001**	0.224	**0.001**	0.278	** < 0.001**	0.000	0.937
**Central 10° VF**	0.185	** < 0.001**	0.175	** < 0.001**	0.234	** < 0.001**	0.167	**0.002**	0.406	** < 0.001**	0.332	** < 0.001**	0.273	** < 0.001**	0.000	0.931
Sup central 10° hemifield	0.063	0.022	0.056	0.031	0.190	**0.001**	0.182	**0.001**	0.258	** < 0.001**	0.126	0.014	0.256	** < 0.001**	0.004	0.655
Inf central 10° hemifield	0.169	** < 0.001**	0.194	** < 0.001**	0.222	** < 0.001**	0.107	0.012	0.355	** < 0.001**	0.318	** < 0.001**	0.236	** < 0.001**	0.002	0.750
**Central 5° VF**	0.183	** < 0.001**	0.155	** < 0.001**	0.228	** < 0.001**	0.203	** < 0.001**	0.331	** < 0.001**	0.280	** < 0.001**	0.325	** < 0.001**	0.002	0.731
Sup central 5° hemifield	0.067	0.018	0.066	0.019	0.184	**0.001**	0.210	** < 0.001**	0.200	**0.002**	0.081	0.053	0.282	** < 0.001**	0.006	0.603
Inf central 5° hemifield	0.167	** < 0.001**	0.188	** < 0.001**	0.194	**0.001**	0.140	**0.004**	0.337	** < 0.001**	0.348	** < 0.001**	0.246	** < 0.001**	0.001	0.836

Bonferroni correction was used for multiple comparisons.* P* value < 0.0083 (0.05/6) was considered significant.

Statistically significant values are shown in bold.

POAG = primary open-angle glaucoma; CS = contrast sensitivity; VFS = visual field sensitivity; VF = visual field; Sup = superior; Inf = inferior.

## Discussion

CS affects visual QOL. Decreased CS may suggest a visual impairment which can have negative effects on the daily lives of patients with glaucoma^4,5,13,14^. Visual QOL is also affected by glaucomatous VF defects. However, different VF areas are required for different visual tasks^11^, indicating that the location of the VF defect, rather than its degree, is a more important predictor of visual QOL^10^. The present study estimated the correlations between CS at two levels and VFS in various hemifield sectors and investigated whether CS is affected by the location of VF damage. The results showed that the CS did not represent the location of VF damage, but it was likely to be affected in the eyes with more severe visual field damage having bi-hemifield VF defect.

Decreased CS in glaucoma has been well documented^1,15–19^. Assessing CS may be more useful in monitoring the progression of functional visual loss than testing VF^1^. Changes in CS of glaucomatous eyes have been detected prior to visible damage to the RNFL, manifest VF defects, or reductions in visual acuity (VA)^15–17^. Decreased CS has been associated with decreased structural thickness measured by OCT^18,19^ and with poorer VF^16^. Few studies, however, have assessed the relationships between locations of VF defects and CS. Although the present study found that there was a generally good correlation between CS and VFS in all sectors, the relationship between CS and VFS was dependent on both the distances at which CS was measured, and the presence of bihemifield VF defects. CS at near distance tended to be more strongly correlated with VFS than CS at far distance. More interestingly, CS did not correlate well with VFS in eyes with VF defects in a single hemifield.

In patients with no VF defects (preperimetric glaucoma), only CS at 0.3 m correlated significantly with VFS in all sectors, whereas CS at 5 m did not correlate with VFS in any sector, indicating that the CS at near distance may better reflect VFS than the CS at far distance in the eyes without VF defects. However, the relationship between CS and VFS was found to differ according to the locations of hemifield VF defects.

In patients with only superior hemifield defects, neither CS at 0.3 m nor at 5 m was significantly associated with VFS in the superior sectors. In contrast, patients with only inferior hemifield defects showed significant correlations between CS and VFS in all but one sector. The present study found that the difference between superior and inferior VFS was larger in eyes with superior than inferior hemifield defects, specifically in the central VF sectors. That is, the reduction in inferior VFS was smaller in eyes with inferior hemifield defects alone than was the reduction in superior VFS in eyes with superior hemifield defects. It is speculated that the decreased VFS in the superior hemifield had no effect on CS, resulting in a poor correlation between the superior VFS and CS in the superior hemifield defect group. Because the reduction of VFS in the inferior hemifield defect group was minimal, it may not have significantly affected the correlation between CS and VFS, resulting in fair correlations in most sectors, including both the superior and inferior sectors. Taken together, these findings suggest that the decreased VFS in a single hemifield may not affect the CS, with the lack of effect likely associated with compensation by the intact hemifield. This hypothesis is supported by the finding that CS did not differ in the groups with inferior and superior hemifield defects, despite their differing degrees of VFS decrease. Although this could have been clarified by sectoral assessment of CS, this assessment could not be performed using the CGT-2000 in this study.

In contrast, eyes with bi-hemifield defects showed good correlation between VFS and CS at 0.3 m in all sectors. This group showed the lowest CS and VFS at the same time, indicating that the decreased CS at 0.3 m was associated with the decreased VFS. However, only the inferior sector, not the superior sector, showed significant correlation between VFS and CS at 5 m. We do not have a decisive answer for this finding, but the relatively better VFS in the inferior field may have compensated for the worse VFS in the superior sector at 5 m. Near visual quality, however, was significantly affected when VFS is decreased in both the superior and inferior hemifields.

Studies have shown significant correlations between structural thicknesses in the inferior area and CS. For example, RNFL thickness in the inferior quadrant was the most strongly correlated with CS score of the superior area^19^, and macular ganglion cell/inner plexiform layer thickness at the inferior macular sectors was significantly associated with CS^18^. The weak association between the superior VF sector (inferior optic nerve/macular region) and CS in the present study was not consistent with previous findings. However, this study cannot be directly compared with other studies because of differences in study design. Patients in the present study were divided into four VF groups based on the location of hemifield VF defects, whereas other studies did not evaluate the location of damage in each participant. In addition, the present study compared two functional measurements, whereas other studies compared structure and function.

This study has several limitations. First, the device used in our study could not measure region specific CS, preventing assessment of regional CS. However, CS loss was rather diffuse throughout the field of vision^19^, suggesting that the global CS may be a sufficient indicator of visual disability. Further study using sector-wise assessment of CS is required for more detailed conclusion. Second, a difference in superior and inferior VFS was not comparable between the groups with superior and inferior VF defects, which could have resulted in biased results. Third, topical anti-hypertensive medication was being administered to all eyes in the present study, which could have affected CS results. Partial reversibility of CS has been observed in glaucoma patients after IOP lowering treatment^20,21^. Moreover, topical medications may have altered the corneal surface, thereby affecting CS results. Fourth, poor reproducibility of the CS testing could be an important confounder, which could not fully be accounted for in this retrospective study.

In conclusion, this study showed that correlations between CS and VFS were dependent on the distances at which CS was measured and on the presence of VF defect in both hemifields. CS may not be an indicator of VFS in eyes with single hemifield VF defects, because the healthy contralateral hemifield may have compensated for defects in the damaged hemifield. In contrast, decreased CS was likely associated with decreased VFS in eyes with bi-hemifield VF defects. Because of the rather diffuse nature of CS loss, the CS test may not substitute VF evaluation. CS, however, may be an indicator of visual disability caused by glaucomatous VF damage, specifically in eyes with VF damage in both hemifields.

## Methods

### Study Subjects

This retrospective analysis included patients with POAG who underwent CS evaluation at the glaucoma clinic of Seoul National University Bundang Hospital between January 2020 and March 2021. The study protocol was approved by the Institutional Review Board of Seoul National University Bundang Hospital, and conformed to the tenets of the Declaration of Helsinki. Informed consent was obtained from all subjects.

All subjects underwent a complete ophthalmic examination, including visual acuity assessment, refraction, slit-lamp biomicroscopy, gonioscopy, Goldmann applanation tonometry, and dilated stereoscopic examination of the optic disc. They also underwent stereo disc photography (EOS D60 digital camera, Canon, Utsunomiya-shi, Tochigi-ken, Japan), measurement of the circumpapillary retinal nerve fiber layer thickness (RNFLT) using spectral-domain optical coherence tomography (SD-OCT; Spectralis OCT, Heidelberg, Engineering, Heidelberg, Germany), standard automated perimetry (24-2 Swedish interactive threshold algorithm and Humphrey Field Analyzer II 750, Carl Zeiss Meditec), and CS (CGT-2000, Takagi Seiko, Nagano-Ken, Japan). Other ophthalmic examinations included measurements of corneal curvature (KR-1800, Topcon), central corneal thickness (CCT; Orbscan II, Bausch & Lomb Surgical, Rochester, NY, USA) and axial length (AXL; IOL Master version 5, Carl Zeiss Meditec, Dublin, CA, USA).

POAG was defined as the presence of an open iridocorneal angle, signs of glaucomatous optic nerve damage (i.e., vertical cup-to-disc ratio ≥ 0.7, asymmetry ≥ 0.2, or the presence of neuroretinal rim thinning, notching, or a splinter hemorrhage), and/or associated VF defects without other ocular diseases or conditions that might cause VF abnormalities^22,23^. A glaucomatous VF defect was defined as (1) glaucoma hemifield test results outside the normal limits; (2) three abnormal points, with a probability of being normal of *P* < 5%, and one point with a pattern deviation of *P* < 1%; or (3) a pattern standard deviation of *P* < 5%. These VF defects were confirmed in two consecutive reliable tests (fixation loss rate ≤ 20% and false-positive and false-negative error rates ≤ 25%)^24^.

To be included in the present study, subjects were required to have a best-corrected visual acuity of at least 20/40, a spherical equivalent between –8.0 and + 3.0 diopters, and a cylinder correction between –3.0 and + 3.0 diopters. Those with a history of ocular surgery other than cataract extraction and glaucoma surgery, other intraocular diseases (e.g., age-related macular degeneration, diabetic retinopathy, or retinal vessel occlusion) or neurological diseases (e.g., pituitary tumors) that could cause VF defects were excluded. Eyes with a history of ocular trauma or uveitis were also excluded. Eyes were excluded if they demonstrated visually significant cataract graded as cortical or nuclear opalescence 1.0 or higher, as classified according to the modification of the Lens Opacities Classification System, version III^25^.

### Contrast sensitivity

CS was measured using a contrast glare tester (CGT-2000), with six target sizes (6.3°, 4.0°, 2.5°, 1.6°, 1.0° and 0.64°) without glare, at distances of both 0.3 m and 5 m. Contrast threshold, defined as the minimum contrast that a subject could see, was measured at 14 levels, from 0.0071 to 0.64. The values were converted to log contrast sensitivity (logCS) for calculation of mean CS and for statistical analysis. During testing, each patient’s pupil was carefully monitored through monitor by the examiner.

### Standard automated perimetry

**Figure 2:**
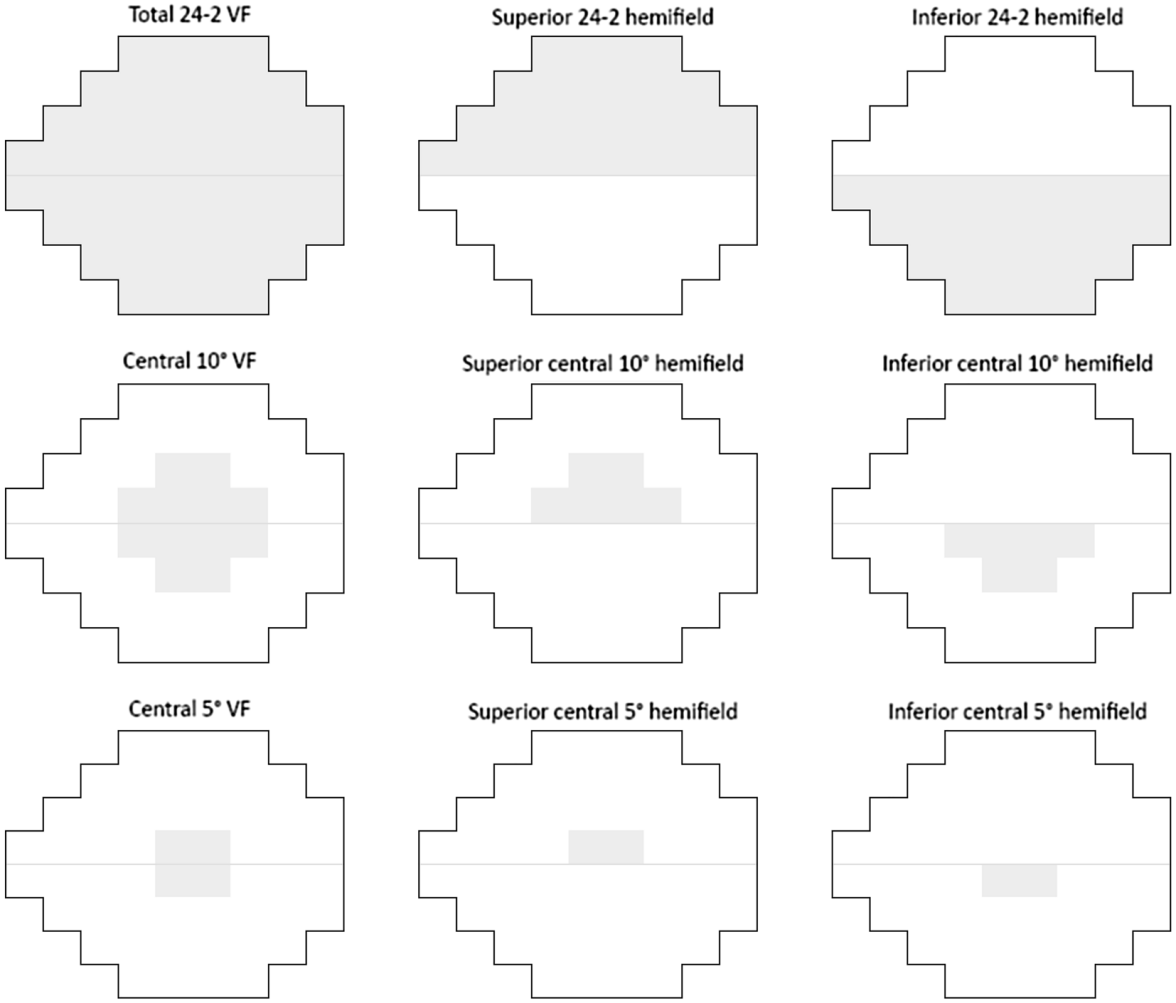
The 24-2 visual field sectors defined to estimate the correlations with contrast sensitivity. VF = visual field.

Standard automated perimetry was performed after CS examinations. Because CS was shown to be closely related to central visual function^18^, sectoral VFS was determined for the central 5-degree VF, the superior central 5-degree hemifield, the inferior central 5-degree hemifield, the central 10-degree VF, the superior central 10-degree hemifield, the inferior central 10-degree hemifield, the inferior 24-2 hemifield, and the superior 24-2 hemifield (Fig. 2). Global VFS was also determined. The anti-logs of the total deviations were determined before averaging and subsequently reconverted to dB values.

The patients were divided into four groups depending on the location of VF defects: those with superior and inferior hemifield defects, those with both, and those with neither (i.e., preperimetric glaucoma).

### Statistical analysis

Correlations of CS with the VFS were assessed using Pearson’s correlation analysis. Between-group comparisons were performed using one-way analysis of variance. All statistical analyses were performed using the Statistical Package for the Social Sciences (version 22.0, SPSS, Chicago, IL, USA). Unless otherwise stated, the data are presented as mean ± SD, with *P* values < 0.05 considered statistically significant.

## Supplementary Information

Supplementary Information.

## Data Availability

The datasets generated during and/or analyzed during the current study are available from the corresponding author on reasonable request.
