# Supplementary material for: Contrast sensitivity in glaucoma patients with visual field defects at different locations
Source: Sci Rep. 2023 Jan 2;13:40. doi: 10.1038/s41598-022-27262-z (PMC9807583; doi:10.1038/s41598-022-27262-z)
Supplement: Supplementary file 1 — Supplementary Information. [file 41598_2022_27262_MOESM1_ESM.docx]

**Supplemental Table 1.** Correlation of contrast and visual field sensitivities with best-corrected visual acuity

|  | **R^2^** | **P-value** |
| --- | --- | --- |
| CS |  |  |
| CS 0.3m | 0.141 | <0.001 |
| CS 5m | 0.235 | <0.001 |
| VFS (dB) |  |  |
| Total 24-2 VF | 0.158 | <0.001 |
| Sup 24-2 hemifield | 0.071 | <0.001 |
| Inf 24-2 hemifield | 0.176 | <0.001 |
| Central 10° VF | 0.216 | <0.001 |
| Sup central 10° hemifield | 0.107 | <0.001 |
| Inf central 10° hemifield | 0.242 | <0.001 |
| Central 5° VF | 0.200 | <0.001 |
| Sup central 5° hemifield | 0.099 | <0.001 |
| Inf central 5° hemifield | 0.239 | <0.001 |

CS = contrast sensitivity; VFS = visual field sensitivity; VF = visual field; Sup = superior; Inf = inferior.
